# Vestibular Rehabilitation Telehealth During the SAEA-CoV-2 (COVID-19) Pandemic

**DOI:** 10.3389/fneur.2021.781482

**Published:** 2022-01-20

**Authors:** Regan G. Harrell, Michael C. Schubert, Sara Oxborough, Susan L. Whitney

**Affiliations:** ^1^Department of Physical Therapy, University of Pittsburgh, Pittsburgh, PA, United States; ^2^Laboratory of Vestibular NeuroAdaptation, Department of Otolaryngology-Head and Neck Surgery and Physical Medicine and Rehabilitation, Johns Hopkins University School of Medicine, Baltimore, MD, United States; ^3^National Dizzy and Balance Center, Bloomington, MN, United States; ^4^Department of Physical Therapy and Otolaryngology, University of Pittsburgh, Pittsburgh, PA, United States

**Keywords:** vestibular rehabilitation, telehealth, dizziness, vertigo, balance, physical therapy

## Abstract

During the COVID-19 pandemic, physical therapists transitioned to provide telehealth in the United States. We sought to determine the experiences of physical therapists delivering telerehabilitation for vestibular disorders including barriers, preferences, and concerns. A survey was created using the results of a focus group and previously published studies. The survey was distributed across social media sites and through email- the link was sent to the orthopedic, neurologic, and geriatric academies of the American Physical Therapy Association list serves. The email was also shared with each of the 50 state chapters of the American Physical Therapy Association. The survey was broken down into five sections: demographic information, physical therapists' general impressions of telehealth, physical therapists' comfort level treating various vestibular diagnoses, and common barriers physical therapists experienced during telehealth sessions. There were 159 completed surveys. More than 80% of physical therapists surveyed agreed that telehealth was an effective platform for vestibular physical therapy. When asked whether physical therapists felt the patient had similar health outcomes with telehealth versus clinic care 68% of physical therapists agreed. For the physical therapists who treated posterior or horizontal canal benign paroxysmal positional vertigo *via* telehealth, more than 50% were comfortable treating these conditions *via* telehealth. In analyzing common peripheral vestibular diagnoses treated *via* telehealth including bilateral vestibular loss, Meniere's disease, and vestibular neuritis more than 75% of the physical therapists reported comfort treating these diagnoses. Similarly, more than 75% of physical therapists who treated central vestibular diagnoses- including mild traumatic brain injury and vestibular migraine- *via* telehealth reported being comfortable treating these diagnoses. Physical therapists reported several barriers to tele healthcare ranging from concerns about testing balance with no caregiver present (94%) to challenges with providing a written home exercise program (33%). Physical therapists report that telehealth is a viable mechanism for providing rehabilitation for persons with balance and vestibular disorders. For common diagnoses, most physical therapists were comfortable treating vestibular disorders *via* telehealth. While barriers remain including maintaining patient safety and being able to complete a thorough vestibular exam, telehealth for vestibular physical therapy services holds promise for the delivery of virtual care.

## Introduction

The severe acute respiratory syndrome coronavirus 2 (SARS-CoV-2, COVID 19) virus upended health care around the world and forever changed how patients converse with and receive care from providers (1, 2). Bruch et al. (2) reported that physicians and psychologists in Germany had a shift to more positive attitudes toward telehealth because of the COVID 19 pandemic. Many clinicians quickly learned to use novel platforms to virtually examine and treat their patients with vestibular disorders and modify how they conducted their physical examination (3). A consensus document that helps guide physician management of persons with acute dizziness provides guidance for history taking and conducting a virtual vestibular physical exam (3). Telehealth visits have successfully been used after otologic surgery safely by surgeons (4).

The vestibular examination generally requires that the clinician be near the patient to view eye movements (1, 5, 6). Physical therapists have attempted to determine the reliability of select internet-based evaluative measures for musculoskeletal conditions (7). In a systematic review and meta-analysis, Cottrell et al. (8) reported that telehealth for musculoskeletal conditions appears to be effective. During the typical physical therapist examination of persons with dizziness an extraocular eye movement examination, the head impulse test, the head shaking test, vestibular ocular cancellation, positional testing, assessment of nystagmus type and direction, and tests of balance are incorporated plus others (5, 6). With vestibular rehabilitation, van Vugt et al. (9) reported that internet based vestibular exercises for persons with chronic vestibular disorders was effective.

The number of telehealth visits with physical therapist exponentially increased during the first 9 months of the pandemic. In the United States, 41 of 50 states prior to the pandemic permitted some form of telehealth by physical therapists in 2018 (10), with 6 states still having no official telehealth legislation in 2021 (11).

The use of telehealth for persons with balance and vestibular disorders may make care more affordable and accessible (12). Persons with complex conditions could be seen by experienced vestibular physical therapists that are not available locally *via* telehealth (13). However, it is important to gain an understanding of which diagnostic conditions physical therapists feel most comfortable treating *via* telehealth. Therefore, the purpose of the study was to understand how vestibular physical therapy changed during the COVID-19 pandemic by transitioning to telehealth. The aims of the survey were to describe physical therapist's general impressions of telehealth, which patient diagnoses physical therapists were comfortable treating, which examination techniques and exercises the physical therapists utilized, and what physical therapists considered as barriers to telehealth.

## Materials and Methods

Prior to the drafting of the Qualtrics survey (Qualtrics, Provo, UT) a review of the current literature on rehabilitation services *via* telehealth was completed. Dahl-Popolizio et al. (14) survey on the provision of occupational therapy experiences during the COVID-19 pandemic was reviewed prior to the development of the physical therapist survey. To determine the categories of questions to ask physical therapists, a focus group of four full time practicing physical therapists with experience delivering vestibular physical therapy *via* telehealth were queried. From the focus group, several themes were determined to be important concepts worth investigating i.e., comfort level with various diagnoses, barriers to treatment.

There were 70 questions broken down into five sections (see Appendix A in Supplementary Material for full survey) to the survey: general impressions about telehealth, comfort level with various diagnoses, examination procedures and exercise usage, barriers to telehealth, and demographic information. At the beginning of the survey, general questions about impressions of the use of telehealth for the delivery of vestibular physical therapy were asked. Physical therapists were asked whether they thought telehealth was an effective platform for delivery of vestibular physical therapy, whether they experienced differences in attendance for scheduled sessions, and whether they believed the patient had a similar health outcome compared with clinic care. Physical therapists were asked to rank their agreement with the survey statements about different diagnoses using a 5-point Likert scale: strongly agree, somewhat agree, neither agree nor disagree, somewhat disagree, and strongly disagree.

Demographic information included age, gender, years of vestibular physical therapy experience, years of telehealth experience, and their primary physical therapy practice clinical location.

Participants were asked if they had treated various vestibular peripheral and central pathologies *via* telehealth including the following diagnoses: Benign paroxysmal peripheral vertigo (BPPV) of the posterior, horizontal, and anterior semicircular canal, bilateral vestibular loss, cerebellar degeneration, Chiari malformation, mild traumatic brain injury (concussion), disequilibrium of aging, labyrinthitis, Mal de Debarquement, Meniere's disease, multiple sclerosis, persistent postural perceptual dizziness, stroke (anterior or posterior inferior cerebellar artery), vestibular migraine, vestibular neuritis, and vestibular schwannoma. If “yes” was selected for either of these diagnoses, participants then completed a 5-point Likert scale rating how comfortable they were treating those diagnosis *via* telehealth: extremely comfortable, somewhat comfortable, neither comfortable nor uncomfortable, somewhat uncomfortable, and extremely uncomfortable.

Participants were asked about whether they were confident with the knowledge generated from various examination techniques and with certain exercises. A list of commonly used tests and measures were provided, listed in **Tables 2A,B** and Appendix A in Supplementary Material. The physical therapist marked all that they felt they were able to effectively provide *via* telehealth. The survey included a free text option for the participant to write in items not included on the provided list of examination procedures and exercises. Similarly, a list of commonly prescribed vestibular exercises was provided, and the participant marked those exercises they felt were effective to deliver *via* telehealth.

For the barriers faced during the telehealth visits section, participants were asked to rate on a 5-point Likert scale how often they experienced the barrier including: always, most of the time, about half of the time, sometimes, or never. Data were sub-categorized into “never” or “that they experienced some barrier.”

The survey was disseminated using Qualtrics software and was approved by the Biomedical IRB of the University of Pittsburgh in Pittsburgh PA, USA. Distribution of the survey occurred through posts on social media sites and email. The email with the survey link was shared with the list serves of the neurologic, geriatric, and orthopedic academies of the American Physical Therapy Association (APTA). The link was also shared with every state physical therapist professional association. Additionally, six state physical therapist associations sent email blasts seeking participation. All completed survey participants acknowledged consent by completing the survey. The survey was open from March 2021–May 2021, since this survey was anonymous there was no follow-up completed.

## Results

One hundred ninety-eight physical therapists completed the survey with 159 surveys analyzed. Forty-one surveys were incomplete and thus were not included in the final analysis. It was not possible to save the survey once it was started, and therefore any incomplete surveys were removed to ensure no one person was represented more than once in the data. One hundred and fifty-nine surveys were analyzed and described with frequencies and percentages. Table 1 shows the demographic information of the respondents including gender, age, and primary location where the physical therapists work. Two subgroups were determined based on years of vestibular experience- 0–10 years of vestibular experience (*n* = 90) and 15+ years of vestibular experience (*n* = 28). Both subgroups demographic information is listed in Table 1.

**Table 1:** Demographic data from the physical therapist who completed the telehealth survey.

	**Total responses *N* = 159**	**Therapists with 0–10 years' experience *N* = 90**	**Therapists with 15+ years' experience *N* = 28**
**Gender**			
Female	133 (84%)	83 (92%)	25 (89%)
Male	22 (14%)	6 (7%)	1 (4%)
Prefer not to say	4 (2%)	1 (1%)	2 (7%)
**Age**			
20–34	58 (36%)	58 (64%)	–
35–54	82 (52%)	32 (36%)	9 (32%)
55–85+	19 (12%)	–	19 (68%)
**Primary location where physical therapist worked**			
Outpatient clinic	135 (85%)	81 (90%)	23 (82%)
Home health	6 (4%)	2 (2%)	3 (10%)
Skilled nursing facility	2 (1%)	1 (1%)	–
School based	2 (1%)	1 (1%)	–
Acute care/hospital	4 (3%)	2 (2%)	1 (4%)
Other^*^	10 (6%)	3 (4%)	1 (4%)

* *Other includes research clinic and academic settings*.

**Figure 1:**
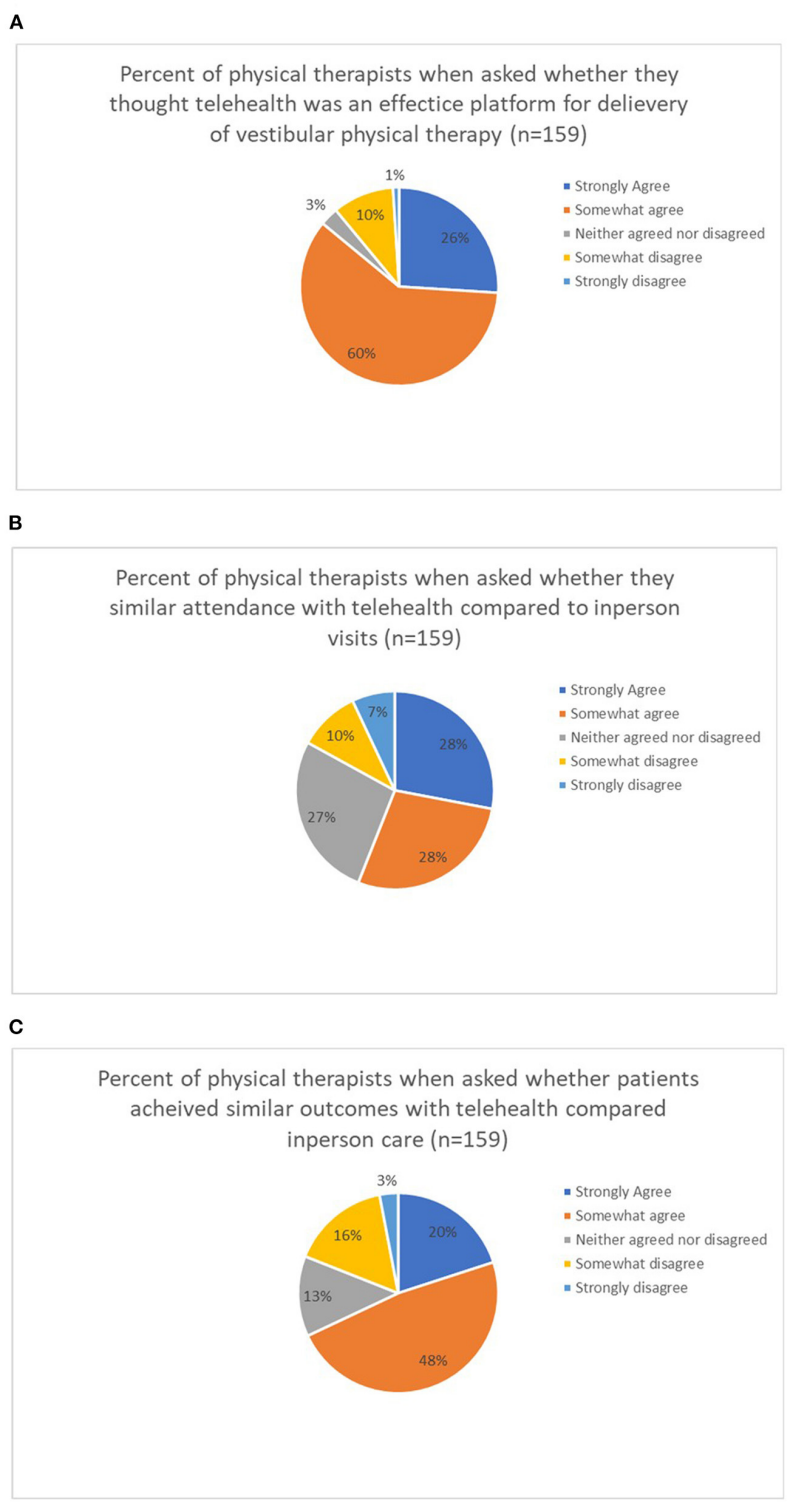
**(A–C)** Percentage of agreement from physical therapists on general impressions of telehealth physical therapy for vestibular physical therapy services.

Participants responses to general impressions are listed in Figure 1. Figure 1A shows the percentage of agreement among participants as it relates to if they felt telehealth was a viable platform for physical therapy services. Figure 1B shows the percentage of agreement as it relates to session attendance and Figure 1C shows the percentage of participants who agreed telehealth produced similar outcomes to face-to-face clinic care.

**Figure 2:**
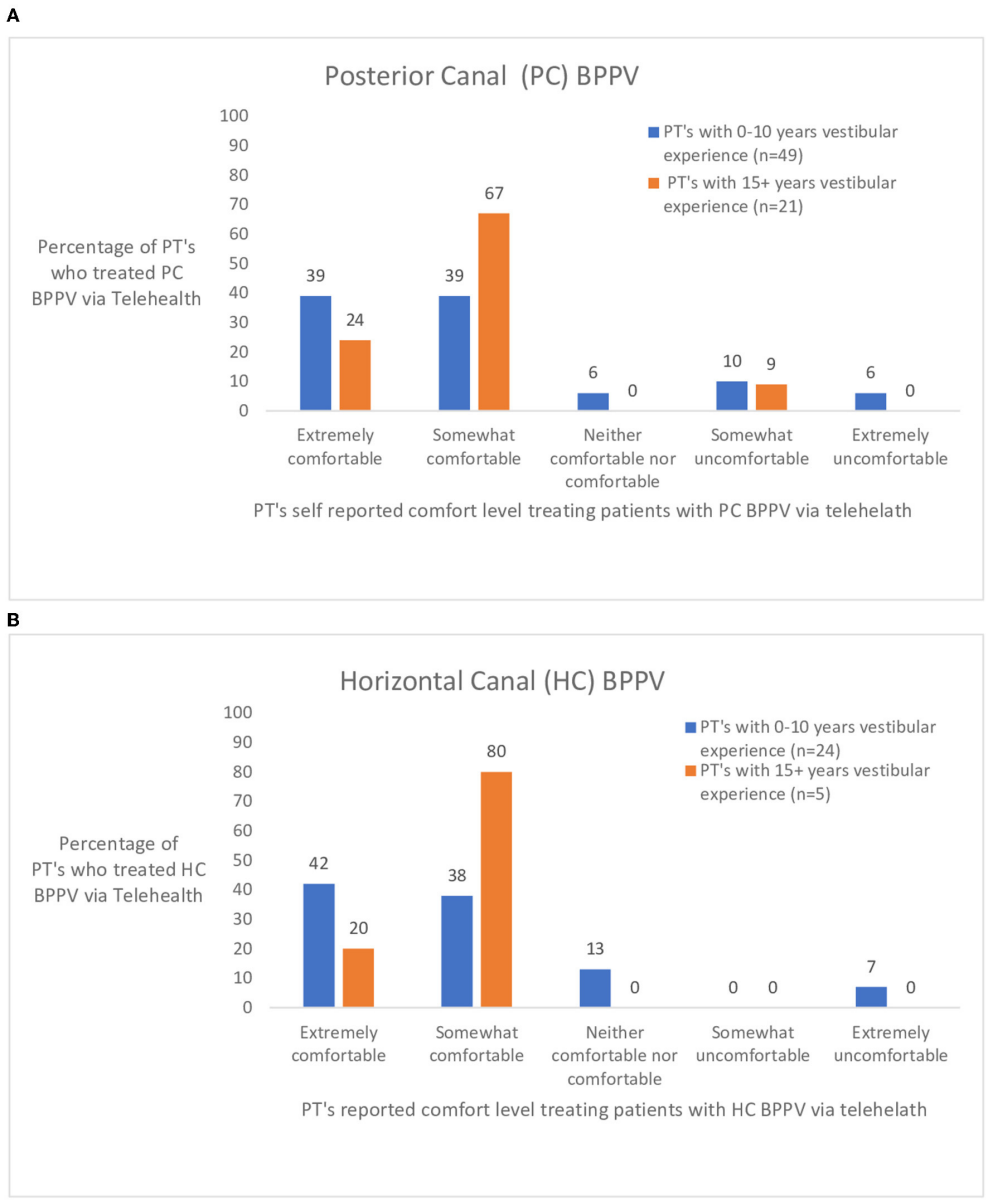
**(A,B)** Rating by physical therapists (PT's) with 0–10 and 15+ years of vestibular experience who treated persons with posterior (PC) and horizontal canal (HC) benign paroxysmal positional vertigo (BPPV) *via* telehealth.

**Figure 3:**
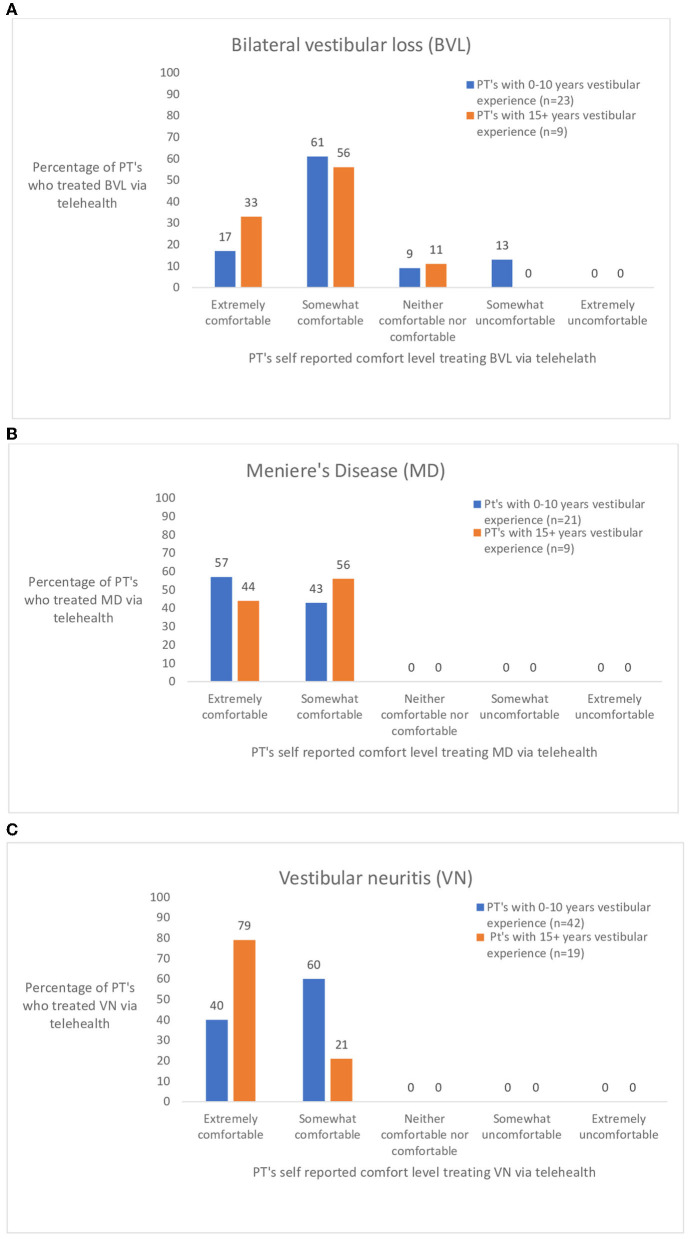
**(A–C)** Ratings by physical therapist (PT's) with 0–10 and 15+ years of vestibular experience who treated persons with common peripheral vestibular diagnoses: bilateral vestibular loss (BVL), Meniere's disease (MD), and vestibular neuritis (VN) *via* telehealth.

**Figure 4:**
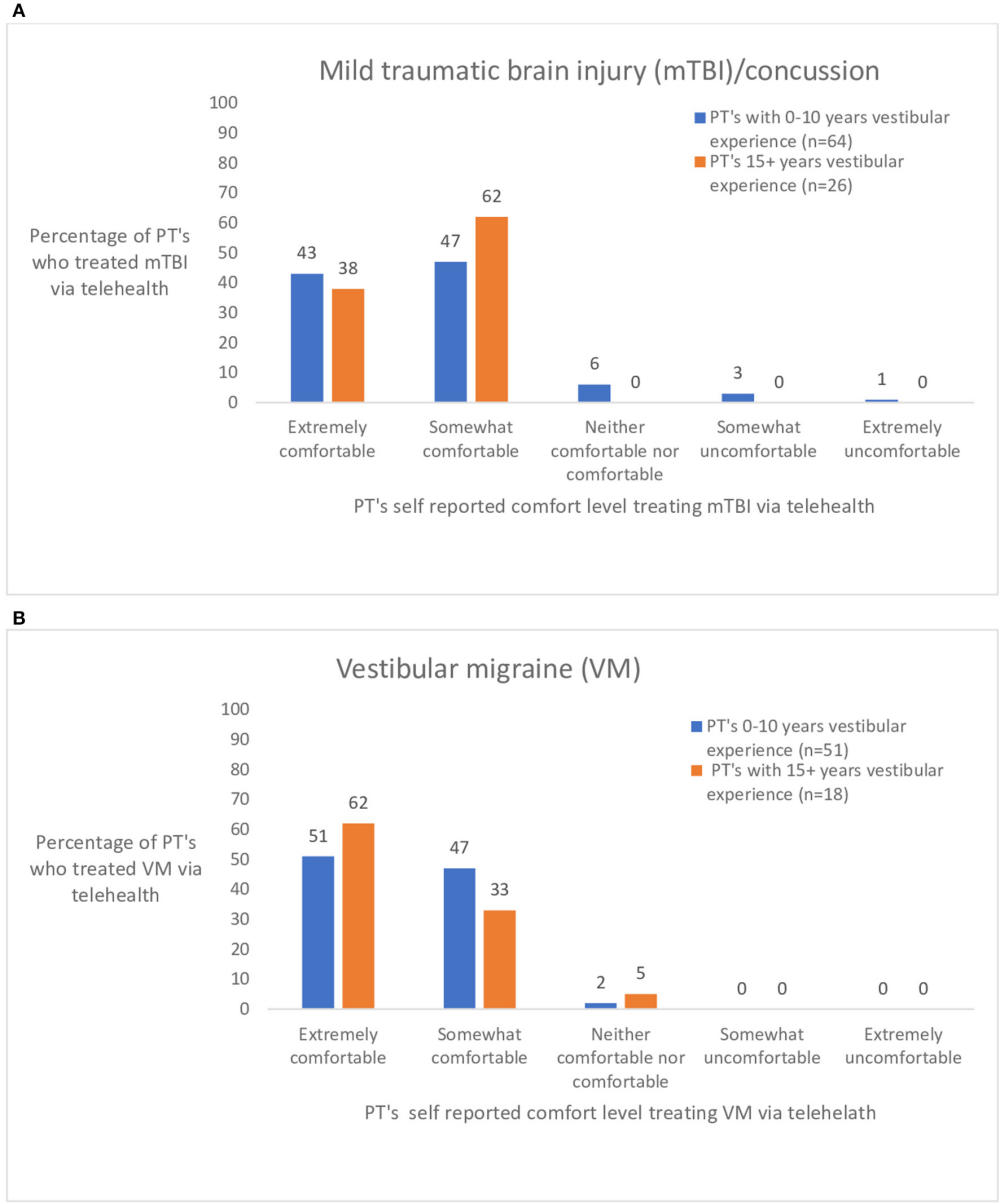
**(A,B)** Ratings by physical therapists (PT's) with 0–10 and 15+ years of vestibular experience who treated persons with common central vestibular diagnoses: mild traumatic brain injury/concussion (mTBI) and vestibular migraine (VM) *via* telehealth. visits were as effective as in-person visits?” (*n* = 159).

In the survey pertaining to various diagnoses seen *via* telehealth, 17 diagnoses were included. Of the 17, the seven mostly commonly seen diagnoses in vestibular clinics were further assessed. The seven diagnoses were chosen based on the frequency that physical therapists treated the condition *via* telehealth. The responses were separated into two groups based on years of vestibular experience- 0–10 years vestibular experience (*n* = 90) and 15+ years of vestibular experience (*n* = 28). Figure 2 shows the ratings of perceived comfort a physical therapist had if they treated someone with posterior canal BPPV (Figure 2A) or horizontal canal BPPV (Figure 2B) *via* telehealth. Figure 2 shows responses to three commonly treated peripheral diagnoses. Figure 3 illustrates the responses of physical therapists self-reported comfort level treating bilateral vestibular loss, Meniere's disease, and vestibular neuritis *via* telehealth. Figure 4 illustrates the responses of physical therapist's self-reported comfort level to common central nervous system vestibular pathologies, vestibular migraine and mild traumatic brain injury.

Along with diagnoses, respondents were asked to rate if they could effectively complete components of the vestibular examination and various vestibular exercises *via* telehealth. The percentage of respondents who felt they could effectively conduct vestibular exam techniques *via* telehealth is included in Table 2A (*n* = 159). Table 2B shows the percentage of respondents who felt they could effectively provide vestibular exercises *via* telehealth (*n* = 159).

**Table 2:** (A,B) Percentage of physical therapist respondents who reported that they could effectively provide vestibular examination techniques and specific exercises *via* telehealth (*n* = 159).

	**Number (Percentage) of respondents**
**Examination**
Cervical range of motion	137 (86%)
Symptom provocation with VORx1	132 (83%)
Smooth pursuit	125 (77%)
Romberg testing	118 (74%)
Saccades	118 (74%)
Home environmental assessment for safety	102 (64%)
Observation of nystagmus in room light	94 (59%)
VOR cancellation	92 (58%)
Clinical test of sensory integration and balance	85 (53%)
Vergence	84 (53%)
Cranial Nerve Function (3,4, & 6)	81 (51%)
Dix Hallpike	81 (51%)
Head shake test	25 (42%)
Hearing screen	25 (42%)
Dynamic visual acuity	25 (42%)
Roll test	62 (39%)
Dynamic gait index	53 (33%)
Cover/uncover test	51 (32%)
Cross cover test	42 (26%)
Gait speed	41 (26%)
Other^*^	16 (10%)
Sensory testing	14 (8%)
Head impulse test	10 (6%)
**Exercise**	
Habituation exercises	149 (94%)
Standing balance exercises- flat surface	147 (93%)
VORx1	147 (93%)
Walking with head turns side to side	121 (76%)
Gaze shift between two targets	118 (74%)
Walking with head turns up/down	114 (72%)
Standing balance exercises- complaint surface	101 (64%)
Saccades	97 (61%)
VORx2	82 (52%)
Walking with dual task	72 (45%)
Walking with quick turns	67 (42%)
Virtual reality exercises	67 (42%)
Remembered or imaginary target exercise	63 (40%)
Walking with eyes closed	39 (25%)
Walking with an obstacle course	35 (22%)
Walking on uneven surfaces	35 (22%)

* *Other includes assessing for ataxia on finger to nose testing and testing for rapid alternating movement*.

**Figure 5:**
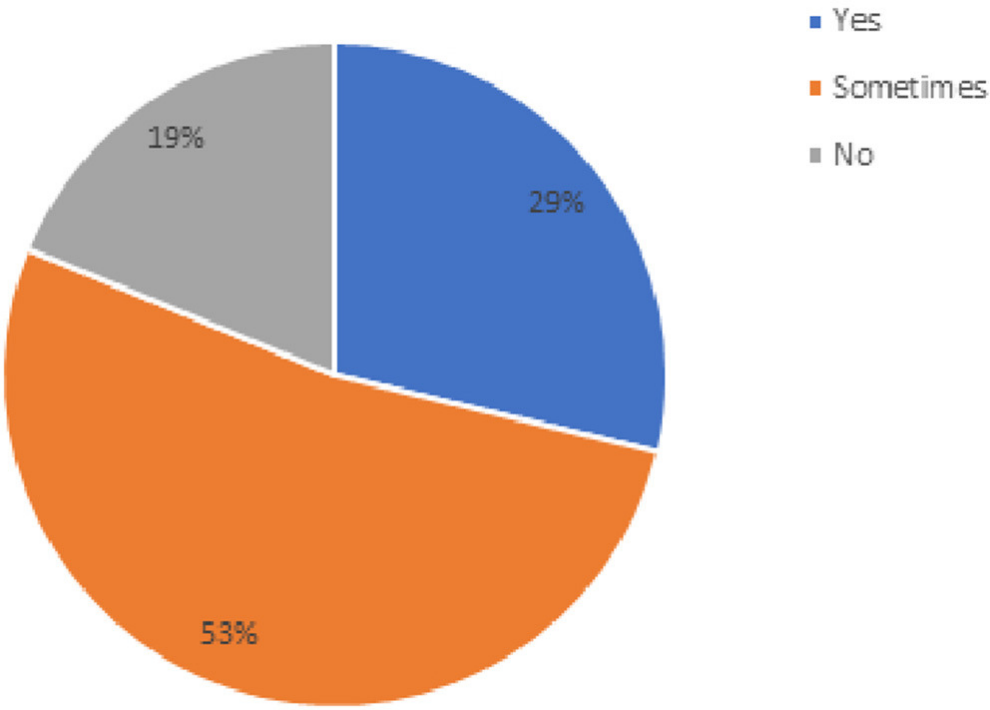
Percentage of physical therapists' responses when asked about “do you have a sense that your telehealth visits were as effective as in-person visits?” (*n* = 159).

The final section of the survey included barriers physical therapists experienced during telehealth sessions. Table 3 lists the percentage of physical therapists who rated that they experienced the barrier either always, most of the time, about half of the time, or some of the time (*n* = 159). The last question on the survey was “Do you have a sense that your telehealth visits were as effective as in-person visits?” and the results are shown in Figure 5.

**Table 3:** Barriers physical therapists reported when completing telehealth vestibular therapy sessions.

**Barrier physical therapist encountered during telehealth visit**	**Number (Percentage) of physical therapist responding**
Concerns about testing balance with no caregiver present	149 (94%)
Bad/inconsistent internet signal	146 (92%)
Equipment set up limiting ability to view patient' body during exam or intervention?	146 (92%)
Patients were not familiar with how to use technology platform	141 (89%)
Difficulty walking with their telecommunications device	130 (82%)
Patient/client had technology incompatible for the visit	125 (79%)
Lack of a caregiver in the home	124 (78%)
Concerns about testing balance with a caregiver present	117 (74%)
Lighting- glare on glasses during the eye exam	104 (66%)
Challenging to provide a written home exercise program	52 (33%)

*All responses except “never” included in the reported percentage below (n = 159)*.

## Discussion

### Demographics

The number of physical therapists in the United States who treat persons with vestibular disorders *via* telehealth is unknown. Werenke et al. (15) reported that 37% of their physical therapists from their outpatient clinics (*n* = 222, 680 patients) provided telehealth care during the second and third quarters of 2020. There are 1,589 members of the Neurology Academy's Vestibular Special Interest (VSIG) group (personal communication, Sara Oxborough, 9/7/2021), but not all VSIG members treated persons *via* telehealth. It is unclear how many of the subjects were recruited from the VSIG vs. from other recruitment mechanisms.

Most of the respondents (66%) had between 6 months to 1 years' experience treating persons *via* telehealth with only 8% having >2 years of experience. Eighty-three percent of the physical therapists had 15 or less years of experience. It is unclear how many physical therapists were required to use telehealth technology to conduct telehealth visits.

Werneke et al. reported that patients who received physical therapy care *via* telehealth were younger and were more likely to live in a metropolitan area (15). The use of technology is a consideration for both the patient and the physical therapist. Sixty-four percent of our physical therapist sample were ≥35 years of age, suggesting that physical therapists were able to adapt to telehealth platforms and the technology requirements to treat patient's virtually (see Table 1). Outpatient physical therapist practices were the most common practice setting (85%), which is not surprising since outpatient practices are the most common employment setting for physical therapists in the US (16).

### General Impressions About Telehealth

Eighty-six percent of the physical therapist respondents felt that telehealth was effective for the delivery of vestibular physical therapy. Cottrell et al. have suggested that telehealth can be effective for the treatment of musculoskeletal conditions (8). Others have reported that patients (*n* = 222,680) were satisfied with their out-patient care for both orthopedic, non-orthopedic, and vestibular physical therapy care *via* telehealth (15). Per their study, the satisfaction ratings of persons seen in the clinic was only 3% higher than those who were treated *via* telehealth (15). Others have reported a 95% patient satisfaction rating in persons seen in outpatient rehabilitation locations (17). Our result of physical therapist satisfaction with telehealth mirrors the satisfaction of patients with their care.

Fifty-six percent of the physical therapists thought that there was enhanced attendance at physical therapy sessions *via* telehealth. Others have reported that attendance might be positively affected with the use of telehealth (18).

Related to whether physical therapists thought that the patient achieved similar outcomes, 68% agree and 19% disagreed and felt that the outcomes were not as good with telehealth. The 19% who reported that they felt that patient had worse outcomes might be related to bandwidth and connection issues, issues related to patients having difficulty with their telecommunication devices, and resistance to change (18).

### Comfort Level With the Diagnoses Seen in Vestibular Physical Therapy *via* Telehealth

BPPV is the most common condition seen in vestibular clinics (19). Physical therapists were generally comfortable using telehealth to treating persons with posterior and horizontal canal BPPV as described by Barreto and Yacovino (1). Barreto and Yacovino (1) utilized cell phones to observe eye movements during the Dix-Hallpike and the roll test. They suggested that it is imperative to utilize telecommunication devices that incorporate both audio and video in the examination and treatment of persons with BPPV *via* telehealth.

However, there were both experienced and less experienced physical therapists who were uncomfortable treating posterior or horizontal canal BPPV *via* telehealth. The survey question did not ask about what lead the physical therapist to be uncomfortable. A possible scenario could be whether the patient had a caregiver at home to assist in the telehealth visit which may have modified their responses as to their level of comfort in treating persons with BPPV.

Persons with BPPV can experience a Tumarkin like event upon resuming the sitting position during the treatment of posterior canal BPPV (20). With older persons, it may be important to have an additional person in the home to avoid a fall after the repositioning maneuver. Barreto and Yacovino suggest that the diagnosis can be made *via* telehealth first, followed by a decision to treat *via* telehealth or in the clinic (1). Clinicians may be more willing to treat persons with BPPV who have previously experienced BPPV in the past (1), as voiced during the focus group meeting. If the person's BPPV symptoms do not resolve with telehealth visits, Shaikh et al. suggest that persons be seen in the clinic (3).

The number of people reporting that they had treated persons with bilateral vestibular loss (BVL) was low and most reported that they were “somewhat comfortable” treating persons with BVL. It is known that persons with BVL fall frequently (21), thus care is required with challenging balance activities in the home.

Unlike BVL, all physical therapists were comfortable with treating persons with Meniere's disease and vestibular neuritis. These two diagnostic groups of the 7 discussed were the only diagnoses where all respondents felt comfortable treating *via* telehealth.

The pandemic reduced sports related concussions by 60% (22). Persons with mild traumatic brain injury were treated later by an average of 26 days compared to pre pandemic time from accident to presentation to the clinic (22). Thus, persons with mild traumatic brain injury seen during the pandemic *via* telehealth may have been more chronic in our survey as well. Generally, participants were comfortable treating persons with mild traumatic brain injury and vestibular migraine *via* telehealth. It may be more difficult to determine if persons post pandemic with mild traumatic brain injury will respond to physical therapy *via* telehealth in a similar manner to those who were seen during the pandemic because of the differences in chronicity.

Overall, physical therapists were comfortable with treating vestibular migraine *via* telehealth.

### Examination Procedures

Per Table 2A, our respondents did not believe that all examination techniques could be effectively administered *via* telehealth. Items endorsed by <30% of the respondents included the cross-cover test, gait speed, coordination testing, sensory testing, and the head impulse test. Green et al. (23) suggested that tests of skew and the alternate cover test could be performed *via* a virtual exam with a cell phone. They suggest that the head impulse test can be performed with active participation of the patient under the direction of the clinician to implement the head impulse test (23). No data is provided to report the reliability or validity of testing the test of skew, nystagmus or head impulse test *via* telehealth (23). The telehealth exam may be hampered by an inadequate frame rate, the ability of the patient or caregiver to hold the phone in the correct position with adequate light to visualize the eyes, or an unstable internet signal (1, 23, 24).

Recording gait speed is a challenge as it is often impossible to accurately determine distances in a person's home to calculate velocity. With cell phones or other technologic devices, it may be difficult to assess coordination and timing of both upper and lower extremity movements.

Sensory testing is a challenge *via* telehealth, yet sensory testing is a vital aspect of the exam that can help guide which balance tests can be performed safely. With loss of distal sensation, performing the Romberg test with eyes closed while a person holds a telecommunication device would not be advised.

Forty-eight percent of the examination procedures utilized in telehealth were rated as not being effective by the physical therapists. There is much work that needs to be done to improve the use of examination procedures commonly utilized by physical therapists in the assessment of persons with balance and vestibular disorders *via* telehealth.

### Exercise in the Home *via* Telehealth

The use of exercises (see Table 2B) had higher efficacy ratings by the physical therapists when compared to the overall ratings of the examination procedures in Table 2A. Although most exercises were endorsed as being able to perform in the home, challenging gait exercises, virtual reality exercises and the remembered or imaginary target exercises had the lowest confidence ratings by the physical therapists. The eye exercises were rated at 50% or greater except for the remembered or imaginary target exercise, which is often difficult to teach and challenging for patients to remember how to perform the exercise correctly regardless of setting.

Participants may have rated the gait exercises low for fear of the patient falling, the difficulty locating a walkway in the home where the device can transmit their gait clearly, or the lack of a caregiver to guard the patient while performing the exercises. It will remain challenging to work on advanced postural skills during gait without having someone nearby to guard the patient. Making decisions to progress based on performance may also be challenging because small nuances in postural control may not be easy to visualize over a telecommunication device. However, remote cell phone monitoring is being done and demonstrates promise in enhancing decision making about fall risk (25, 26). The in-home utilization of cell phones to monitor sit to stand abilities and walking will assist in future telehealth decision making (25, 26). In summary, examination of the client in the home appears to be more challenging than providing exercises for rehabilitation *via* telehealth.

### Barriers to Telehealth

As reported in Table 3, the primary concern was treating the patient without a caregiver present followed by an inconsistent internet signal and the ability to see the correct body part to make a sound clinical judgement (3, 12). Others have reported that familiarity with the use of technology is a barrier, as did our respondents (18). There were concerns about the ability of persons to walk with a telecommunication device if their gait was impaired and concerns about a person's balance. The other concern was that with the lighting (too much or too little) the physical therapist was challenged in conducting an adequate exam to make clinical decisions (3). Although not asked in our survey, persons who are hearing challenged may have more difficulty communicating *via* telecommunications (12, 27) and is something to consider with persons with both vestibular and audiologic impairments.

Only 29% of the physical therapists felt that telehealth was as effective as in person care (see Figure 4). With enhanced video streaming, greater comfort with the technology, and virtual mechanisms to better assess posture and gait, these effectiveness ratings may improve with time.

### Limitations

The survey was active during March-May 2021. It is impossible to know how robust our response rate was since it is unknown how many physical therapists in the United States treated persons *via* telehealth with balance and vestibular disorders. The response rate of 198 is most likely low. However, Dahl-Popolizio et al. (14) had 230 surveys returned about occupational therapists' experiences with telehealth from 137,000 members. The study was mainly distributed through state chapters, academies of the American Physical Therapy Association and social media. Our methods of survey distribution may have limited access to the survey and could have potentially biased sample. Another limitation to the study includes the inability to complete any follow-up after the initial survey. We were unable to determine if physical therapist's perception of telehealth during the pandemic changed as the pandemic progressed.

Only 8% of the respondents had 2 years or more of experience conducting telehealth visits, yet most of the physical therapists were positive in their responses. It is possible that only those physical therapists who liked performing telehealth visits responded to the survey request biasing the findings. Not all out-patient physical therapists performed telehealth during the shutdowns in the United States (15). It appears that some form of telehealth will continue in most areas of the world long after the pandemic has stabilized (28–34).

### The Future of Telehealth With Vestibular Physical Therapy

It appears that there is a promising future for the telehealth delivery of vestibular physical therapy. Improved technology may assist with some of the technological issues revealed by this study (i.e., eye movement examination). The assessment of balance and postural control will be a more challenging issue for telehealth users, although with recent advances in technology the examination of postural control and gait is improving. Developing guidelines or rules to help determine when it is safe to test people in challenging positions will need to be determined and shared to prevent falls during telehealth visits. Overall physical therapist satisfaction appeared high with telehealth, yet physical therapists continued to feel that person to person visits yielded more effective visits.

## Data Availability Statement

The original contributions presented in the study are included in the article/Supplementary Material, further inquiries can be directed to the corresponding author/s.

## Ethics Statement

The studies involving human participants were reviewed and approved by STUDY21020099. The patients/participants provided their written informed consent to participate in this study.

## Author Contributions

All authors listed have made a substantial, direct, and intellectual contribution to the work and approved it for publication.

## Conflict of Interest

The authors declare that the research was conducted in the absence of any commercial or financial relationships that could be construed as a potential conflict of interest.

## Publisher's Note

All claims expressed in this article are solely those of the authors and do not necessarily represent those of their affiliated organizations, or those of the publisher, the editors and the reviewers. Any product that may be evaluated in this article, or claim that may be made by its manufacturer, is not guaranteed or endorsed by the publisher.
