# Supplementary material for: Vestibular Rehabilitation Telehealth During the SAEA-CoV-2 (COVID-19) Pandemic
Source: Front Neurol. 2022 Jan 20;12:781482. doi: 10.3389/fneur.2021.781482 (PMC8811028; doi:10.3389/fneur.2021.781482)
Supplement: Supplementary file 1 [file Table_1.DOCX]

**Appendix A**

**Vestibular Rehab in Telehealth**

Q1) Welcome to the research study! You are being asked to participate since you have some experience with treating people with balance and vestibular disorders via telehealth.  We are especially interested in learning about which evaluative measures and interventions worked well or did not work well with telehealth technology. In addition, we are interested in which patient/client diagnoses are safer/easier to treat via telehealth.  We hope that the knowledge and opinions that you share will help to shape telehealth care for the future for persons with balance and vestibular disorders.  Please be assured that your responses will be kept completely confidential. 
 
The study should take you less than 10 minutes to complete. Your participation in this research is voluntary. You have the right to withdraw at any point during the study, for any reason, and without any prejudice. There are no risks or benefits from completing this survey, All responses to this survey are confidential.  By clicking the button below, you acknowledge that your participation in the study is voluntary, you are 18 years of age, and that you are aware that you may choose to terminate your participation in the study at any time and for any reason. 
 
 
If you would like more information about the survey, please contact the primary investigator: Susan L. Whitney, DPT, PhD, NCS, FAPTA at whitney@pitt.edu Affiliation: University of Pittsburgh

- I consent to participate in this survey  (1)
- I do not consent to participate in this survey  (2)

Q2 **The questions below are being asked so that we can understand where and how you performed telehealth.** 
Which setting best describes your vestibular physical therapy practice?

- Outpatient  (1)
- Home Health/home care  (2)
- Skilled Nursing Facility  (3)
- School Based  (4)
- Acute Care/hospital  (5)
- Other  (6) ________________________________________________

Q3) What platform do you like best for your telehealth vestibular physical therapy practice?

- Adobe Connect  (1)
- Curatess  (2)
- Doxy  (3)
- Facebook Messenger  (4)
- FaceTIme  (5)
- Google Duo  (6)
- Google Meet  (7)
- InTouch  (8)
- Microsoft Teams  (9)
- Zoom  (10)
- Skype  (11)
- Theraplatform  (12)
- Webex  (13)
- Whatsapp  (14)
- Other- please specificy  (15) ________________________________________________

**The questions below are being asked to better understand how you felt about the delivery of telehealth for people with balance and vestibular disorders in your practice.** 
Q4 In my professional opinion, telehealth is an effective platform for the delivery of vestibular and balance physical therapy services:

- Strongly agree  (1)
- Somewhat agree  (2)
- Neither agree nor disagree  (3)
- Somewhat disagree  (4)
- Strongly disagree  (5)

Q5 I had fewer no-shows for vestibular telehealth visits than I usually have for in-person visits

- Strongly agree  (1)
- Somewhat agree  (2)
- Neither agree nor disagree  (3)
- Somewhat disagree  (4)
- Strongly disagree  (5)

Q6 I was able to achieve established patient goals in vestibular rehab via telehealth

- Strongly agree  (1)
- Somewhat agree  (2)
- Neither agree nor disagree  (3)
- Somewhat disagree  (4)
- Strongly disagree  (5)

Q7 I achieved similar health outcomes for vestibular rehab using telehealth as I would have expected in person.

- Strongly agree  (1)
- Somewhat agree  (2)
- Neither agree nor disagree  (3)
- Somewhat disagree  (4)
- Strongly disagree  (5)

Q8 Patients were satisfied telehealth as a delivery platform for vestibular rehab

- Strongly agree  (1)
- Somewhat agree  (2)
- Neither agree nor disagree  (3)
- Somewhat disagree  (4)
- Strongly disagree  (5)

Q9 Caregivers were satisfied with telehealth as a delivery platform for vestibular rehab

- Strongly agree  (1)
- Somewhat agree  (2)
- Neither agree nor disagree  (3)
- Somewhat disagree  (4)
- Strongly disagree  (5)

Q10 Have you treated patients via telehealth with: **Benign paroxysmal positional vertigo (BPPV): Posterior Canal**

- Yes  (1)
- No  (2)

Q11 How comfortable did you feel treating someone with Benign paroxysmal positional vertigo (BPPV): Posterior Canal via telehealth?

- Extremely comfortable  (1)
- Somewhat comfortable  (2)
- Neither comfortable nor uncomfortable  (3)
- Somewhat uncomfortable  (4)
- Extremely uncomfortable  (5)

Q12 Have you treated patients via telehealth with: Benign paroxysmal positional vertigo (BPPV): Horizontal Canal

- Yes  (1)
- No  (2)

Q13 How comfortable did you feel treating someone with Benign paroxysmal positional vertigo (BPPV): Horizontal Canal via telehealth?

- Extremely comfortable  (1)
- Somewhat comfortable  (2)
- Neither comfortable nor uncomfortable  (3)
- Somewhat uncomfortable  (4)
- Extremely uncomfortable  (5)

Q14 Have you treated patients via telehealth with: Benign paroxysmal positional vertigo (BPPV): Anterior Canal

- Yes  (1)
- No  (2)

Q15 How comfortable did you feel treating someone Benign paroxysmal positional vertigo (BPPV): Anterior Canal via telehealth?

- Extremely comfortable  (1)
- Somewhat comfortable  (2)
- Neither comfortable nor uncomfortable  (3)
- Somewhat uncomfortable  (4)
- Extremely uncomfortable  (5)

Q16 Have you treated patients via telehealth with: Bilateral vestibular loss

- Yes  (1)
- No  (2)

Q17 How comfortable did you feel treating someone with **b**ilateral vestibular loss via telehealth?

- Extremely comfortable  (1)
- Somewhat comfortable  (2)
- Neither comfortable nor uncomfortable  (3)
- Somewhat uncomfortable  (4)
- Extremely uncomfortable  (5)

Q18 Have you treated patients via telehealth with: Cerebellar degeneration

- Yes  (1)
- No  (2)

Q19 How comfortable did you feel treating someone with Cerebellar degeneration via telehealth?

- Extremely comfortable  (1)
- Somewhat comfortable  (2)
- Neither comfortable nor uncomfortable  (3)
- Somewhat uncomfortable  (4)
- Extremely uncomfortable  (5)

Q20 Have you treated patients via telehealth with: **Chiari malformation**

- Yes  (1)
- No  (2)

Q21 How comfortable did you feel treating someone with Chiari malformation via telehealth?

- Extremely comfortable  (1)
- Somewhat comfortable  (2)
- Neither comfortable nor uncomfortable  (3)
- Somewhat uncomfortable  (4)
- Extremely uncomfortable  (5)

Q22 Have you treated patients via telehealth with: Concussion/ mild brain injury

- Yes  (1)
- No  (2)

Q23 How comfortable did you feel treating someone with Concussion/ mild brain injury via telehealth?

- Extremely comfortable  (1)
- Somewhat comfortable  (2)
- Neither comfortable nor uncomfortable  (3)
- Somewhat uncomfortable  (4)
- Extremely uncomfortable  (5)

Q24 Have you treated patients via telehealth with: Disequilibrium of aging

- Yes  (1)
- No  (2)

Q25 How comfortable did you feel treating someone with Disequilibrium of aging via telehealth?

- Extremely comfortable  (1)
- Somewhat comfortable  (2)
- Neither comfortable nor uncomfortable  (3)
- Somewhat uncomfortable  (4)
- Extremely uncomfortable  (5)

Q26 Have you treated patients via telehealth with: Labyrinthitis

- Yes  (1)
- No  (2)

Q27 How comfortable did you feel treating someone with Labyrinthitis via telehealth?

- Extremely comfortable  (1)
- Somewhat comfortable  (2)
- Neither comfortable nor uncomfortable  (3)
- Somewhat uncomfortable  (4)
- Extremely uncomfortable  (5)

Q28 Have you treated patients via telehealth with: Mal de Debarquement

- Yes  (1)
- No  (2)

Q29 How comfortable did you feel treating someone with Mal de Debarquement  via telehealth?

- Extremely comfortable  (1)
- Somewhat comfortable  (2)
- Neither comfortable nor uncomfortable  (3)
- Somewhat uncomfortable  (4)
- Extremely uncomfortable  (5)

Q30 Have you treated patients via telehealth with: Meniere's disease

- Yes  (1)
- No  (2)

Q31 How comfortable did you feel treating someone with Meniere's disease via telehealth?

- Extremely comfortable  (1)
- Somewhat comfortable  (2)
- Neither comfortable nor uncomfortable  (3)
- Somewhat uncomfortable  (4)
- Extremely uncomfortable  (5)

Q32 Have you treated patients via telehealth with: Multiple Sclerosis

- Yes  (1)
- No  (2)

Q33 How comfortable did you feel treating someone with Multiple Sclerosis via telehealth?

- Extremely comfortable  (1)
- Somewhat comfortable  (2)
- Neither comfortable nor uncomfortable  (3)
- Somewhat uncomfortable  (4)
- Extremely uncomfortable  (5)

Q34 Have you treated patients via telehealth with: Persistent postural-perceptual dizziness (PPPD)

- Yes  (1)
- No  (2)

Q35 How comfortable did you feel treating someone with Persistent postural-perceptual dizziness (PPPD) via telehealth?

- Extremely comfortable  (1)
- Somewhat comfortable  (2)
- Neither comfortable nor uncomfortable  (3)
- Somewhat uncomfortable  (4)
- Extremely uncomfortable  (5)

Q36 Have you treated patients via telehealth with: Stroke (either anterior or posterior inferior cerebellar artery stroke)

- Yes  (1)
- No  (2)

Q37 How comfortable did you feel treating someone with Stroke (either anterior or posterior inferior cerebellar artery stroke) via telehealth?

- Extremely comfortable  (1)
- Somewhat comfortable  (2)
- Neither comfortable nor uncomfortable  (3)
- Somewhat uncomfortable  (4)
- Extremely uncomfortable  (5)

Q38 Have you treated patients via telehealth with: Vestibular migraine

- Yes  (1)
- No  (2)

Q39 How comfortable did you feel treating someone with Vestibular migraine via telehealth?

- Extremely comfortable  (1)
- Somewhat comfortable  (2)
- Neither comfortable nor uncomfortable  (3)
- Somewhat uncomfortable  (4)
- Extremely uncomfortable  (5)

Q40 Have you treated patients via telehealth with: Vestibular Neuritis

- Yes  (1)
- No  (2)

Q41 How comfortable did you feel treating someone with Vestibular Neuritis via telehealth?

- Extremely comfortable  (1)
- Somewhat comfortable  (2)
- Neither comfortable nor uncomfortable  (3)
- Somewhat uncomfortable  (4)
- Extremely uncomfortable  (5)

Q42 Have you treated patients via telehealth with: Vestibular schwannoma

- Yes  (1)
- No  (2)

Q43 How comfortable did you feel treating someone with Vestibular schwannoma via telehealth?

- Extremely comfortable  (1)
- Somewhat comfortable  (2)
- Neither comfortable nor uncomfortable  (3)
- Somewhat uncomfortable  (4)
- Extremely uncomfortable  (5)

Q44 **The following questions ask you about your vestibular exam and interventions** 
Which of the following examination techniques did you feel you could effectively perform during telehealth visits? Check all that apply

- Cervical range of motion  (1)
- Clinical Test of Sensory Integration and Balance  (2)
- Cover/uncover test  (3)
- Cranial Nerve function (3,4, &6)  (4)
- Cross cover test  (5)
- Dix Hallpike  (6)
- Head Shake Test  (7)
- Hearing Screen  (8)
- Home environmental assessment for safety  (9)
- Dynamic Gait Index  (10)
- Dynamic Visual Acuity  (11)
- Head Impulse test  (12)
- Gait Speed  (13)
- Observation of nystagmus in room light  (14)
- Roll Test  (15)
- Romberg testing  (16)
- Saccades  (17)
- Sensory testing  (18)
- Smooth pursuit  (19)
- Vergence  (20)
- VOR cancellation  (21)
- Symptom provocation with VORx1  (22)
- Other  (23) ________________________________________________

Q45 Which of the following exercises did you provide during telehealth? Check all that apply 
Habituation exercises  (1)

- Remembered or imaginary target exercise  (2)
- Gaze shift between two targets  (3)
- Saccades  (4)
- Smooth pursuits  (5)
- Standing balance exercises- flat surface  (6)
- Standing balance exercises- complaint surfaces  (7)
- Vergence exercises  (8)
- Virtual reality exercises  (9)
- VORx1  (10)
- VORx2  (11)
- Walking with eyes closed  (12)
- Walking with dual tasking  (13)
- Walking with head turns  (14)
- Walking with head moving up/down  (15)
- Walking with an obstacle course  (16)
- Walking with quick turns  (17)
- Walking on uneven surfaces  (18)
- Other  (19) ________________________________________________

Q46 What comorbidities were worrisome to you as you developed your intervention program?

- None  (4)
- Please list below  (5) ________________________________________________

Q47 How often did you experience the following challenges with delivering your telehealth care?

Bad/inconsistent internet signal

- Always  (1)
- Most of the time  (2)
- About half the time  (3)
- Sometimes  (4)
- Never  (5)

Q48 How often did you experience the following challenges with delivering your telehealth care?  
Patient/client had technology incompatible for the visit

- Always  (1)
- Most of the time  (2)
- About half the time  (3)
- Sometimes  (4)
- Never  (5)

Q49 How often did you experience the following challenges with delivering your telehealth care? 
Equipment set up limiting ability to view patient' body during exam or intervention?

- Always  (9)
- Most of the time  (10)
- About half the time  (11)
- Sometimes  (12)
- Never  (13)

Q50 How often did you experience the following challenges with delivering your telehealth care?

Patients were not familiar with how to use technology platform

- Always  (1)
- Most of the time  (2)
- About half the time  (3)
- Sometimes  (4)
- Never  (5)

Q51 How often did you experience the following challenges with delivering your telehealth care?  
Lack of a caregiver in the home

- Always  (1)
- Most of the time  (2)
- About half the time  (3)
- Sometimes  (4)
- Never  (5)

Q52 How often did you experience the following challenges with delivering your telehealth care?  
Challenging to provide a written home exercise program

- Always  (1)
- Most of the time  (2)
- About half the time  (3)
- Sometimes  (4)
- Never  (5)

Q53 How often did you experience the following challenges with delivering your telehealth care?  
Concerns about testing balance with a caregiver present

- Always  (1)
- Most of the time  (2)
- About half the time  (3)
- Sometimes  (4)
- Never  (5)

Q54 How often did you experience the following challenges with delivering your telehealth care?  
Concerns about testing balance with no caregiver present

- Always  (1)
- Most of the time  (2)
- About half the time  (3)
- Sometimes  (4)
- Never  (5)

Q55 How often did you experience the following challenges with delivering your telehealth care?  
Difficulty walking with their telecommunications device

- Always  (1)
- Most of the time  (2)
- About half the time  (3)
- Sometimes  (4)
- Never  (5)

Q56 How often did you experience the following challenges with delivering your telehealth care?

Lighting- glare on glasses during the eye exam

- Always  (1)
- Most of the time  (2)
- About half the time  (3)
- Sometimes  (4)
- Never  (5)

Q57 Did you experience difficulty with documenting your visit?

- Yes  (3)
- Sometimes  (4)
- No  (5)

Q58 Did you experience difficulty making follow up or new appointments?

- Yes  (3)
- Sometimes  (4)
- No  (5)

Q59 Did you experience difficulty sending communication to the patient after/before the visit?

- Yes  (3)
- Sometimes  (4)
- No  (5)

Q60 Do you have a sense that your telehealth visits were as effective with in-person visits?

- Yes  (3)
- Sometimes  (4)
- No  (5)

Q61 Did you provide telehealth vestibular rehabilitation to out of state patients?

- Yes  (1)
- No  (2)

Q62 What were the positive aspects of telehealth? Check all that apply?

- Good for patients that live far from the clinic  (1)
- Better than no care  (2)
- Other  (3) ________________________________________________

Q63 Did telehealth provide unique information that an in-person visit would not?  If yes, please identify

- Yes  (21) ________________________________________________
- No  (22)

Q64 Is there anything that you would like to share with us about your telehealth experience

________________________________________________________________

Q65 **Please complete the demographic data to help us describe the sample:** 
Gender

- Male  (1)
- Female  (2)
- Non-binary / third gender  (3)
- Prefer not to say  (4)

Q66 Age

- 20- 24  (11)
- 25 - 34  (12)
- 35 - 44  (13)
- 45 - 54  (14)
- 55 - 64  (15)
- 65 - 74  (16)
- 75 - 84  (17)
- 85 or older  (18)

Q67 Years of experience as a physical therapist

- 0-5 years  (1)
- 5-10 years  (2)
- 10-15 years  (3)
- 15-20 years  (4)
- 25+ years  (5)

Q68 Years of experience in vestibular physical therapy

- 0-5 years  (1)
- 5-10 years  (2)
- 10-15 years  (3)
- 15-20 years  (4)
- 20+ years  (5)

Q69 Experience using telehealth with persons with vestibular disorders

- <6 months  (1)
- 6 months- 1 year  (2)
- 1-2 years  (3)
- > 2 years  (4)

Q70 In which state do you currently reside?

▼ Alabama (1) ... Other (54)

Q71 What country do you reside in?
